# Echocardiographic Measures of Diastolic Function Are Preload Dependent during Triggered Positive Pressure Ventilation: A Controlled Crossover Study in Healthy Subjects

**DOI:** 10.1155/2012/703196

**Published:** 2012-09-25

**Authors:** Peter Juhl-Olsen, Christian Alcaraz Frederiksen, Johan Fridolf Hermansen, Carl-Johan Jakobsen, Erik Sloth

**Affiliations:** ^1^Department of Anaesthesiology and Intensive Care, Aarhus University Hospital, Skejby, Brendstrupgaardsvej 100, 8200 Aarhus N, Denmark; ^2^Institute of Clinical Medicine, Aarhus University, 8000 Aarhus C, Denmark

## Abstract

*Background*. The use of echocardiography in intensive care settings impacts decision making. A prerequisite for the use of echocardiography is relative resistance to changes in volume status and levels of positive pressure ventilation (PPV). Studies on indices of diastolic function report conflicting results with regard to dependence on volume status. Evidence is scarce on PPV. *Methods*. Ten healthy subjects were exposed to 6 levels of positive end-expiratory pressure (PEEP) and pressure support (PS) following a baseline reading. All ventilator settings were performed at three positions: horizontal, reverse-Trendelenburg, and Trendelenburg. Echocardiography was performed throughout. * Results*. During spontaneous breathing, early diastolic transmitral velocity (*E*) changed with positioning (*P* < 0.001), whereas early diastolic velocity of the mitral annulus (*e*′) was independent (*P* = 0.263). With PPV, *E* and *e*′ proved preload dependent (*P *  values < 0.001). Increases in PEEP, PS, or a combination influenced *E* and *e*′ in reverse-Trendelenburg- and horizontal positions, but not in the Trendelenburg position. *Discussion*. The change towards preload dependency of *e*′ with PPV suggests that PPV increases myocardial preload sensitivity. The susceptibility of *E* and *e*′ to preload changes during PPV discourages their use in settings of volume shifts or during changes in ventilator settings. * Conclusion*. Positioning and PPV affect *E* and *e*′.

## 1. Introduction

**Figure 1:**
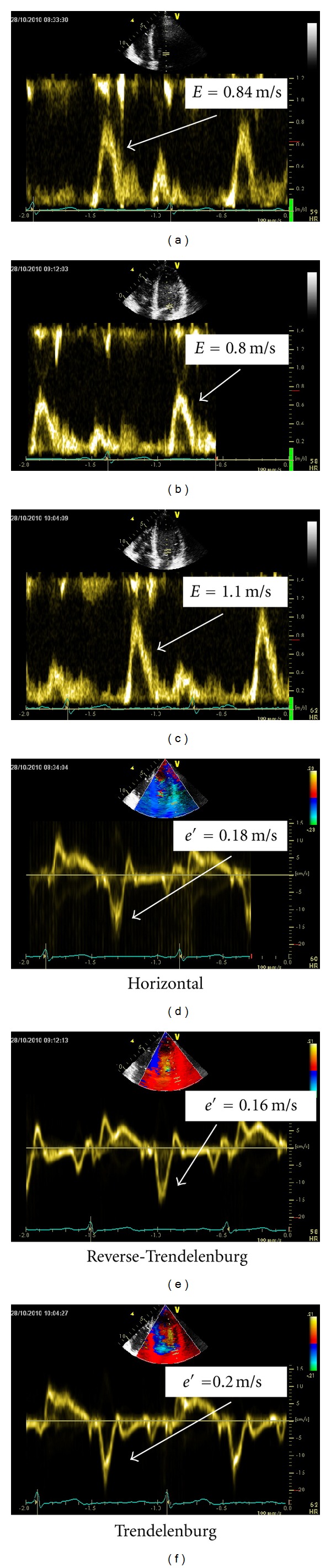
Examples of echocardiographic indices of diastolic function in the horizontal position, reverse-Trendelenburg position, and the Trendelenburg position during positive pressure ventilation. *e*′: peak early diastolic velocity of the lateral mitral annulus, obtained with tissue Doppler. *E*: peak early transmitral blood flow.

The use of echocardiography in perioperative and intensive care settings is feasible and impacts substantially on therapeutic decision making [1–3]. Echocardiographic evaluation involves quantification of systolic and diastolic function. Diastolic function is best assessed using a combination of the mitral valve inflow velocities, *E* (early) and *A* (atrial), and the early diastolic velocity of the mitral valve annuli, *e*′ [4, 5] (Figure 1).

Intensive care therapy often includes aggressive fluid therapy, hemodialysis, or positive pressure ventilation (PPV). Thus, a prerequisite for using echocardiographic measures of diastolic function for primary or repeated evaluation is a relative independence of volume shifts and changes in ventilator settings [6].

Previous studies have concluded that *E* and *A* are sensitive to preload changes [7, 8]. However, with regard to *e*′, clinical and animal studies addressing the issue have shown ambiguous results [9–16]. The composite ratio, *E*/*e*′, has also yielded conflicting results [9, 13, 15].

The use of PPV influences preload, afterload and contractility and may therefore affect indices of diastolic function [17]. Accordingly, studies suggest that a high positive end-expiratory pressure (PEEP) decreases peak *E* velocity [18, 19]. Few studies incorporate *e*′ and *E*/*e*′, and these suggest that both *e*′ an *E*/*e*′ may be insensitive to alterations in PEEP [20, 21]. To our knowledge, the effects of increasing pressure support (PS) or combinations of PEEP and PS have not been clarified. 

The aim of this study was to evaluate the effects of alternating preload, obtained by varying positioning, and of varying PEEP and PS on transmitral Doppler and tissue Doppler measures of diastolic function (Figure 1). 

We hypothesized that *e*′ would be independent of positioning and isolated increases in PEEP, PS, or a combination of PEEP and PS. In addition, we hypothesized that *E* was not independent of these factors.

## 2. Materials and Methods

### 2.1. Design

The study was a controlled, crossover study approved by the Central Denmark Region Committee of Biomedical Research Ethics (journal no. M-ÅA-20060164) and carried out in compliance with the Helsinki Declaration. Written informed consent was obtained from all participants. 

Ten healthy subjects aged 23–32 years were enrolled after an initial screening ensuring optimal echocardiographic image quality. All were thoroughly trained in compliance with triggered PPV at all ventilator settings.

### 2.2. Study Protocol

Subjects were initially positioned horizontally for a baseline measurement at neutral preload and afterwards connected to a positive pressure ventilator without leaks (Bipap Vision, Respironics, Pennsylvania, USA) using a tight-fitting mask. Trigger level was set at −3 cmH_2_O. A fixed sequence of the following ventilator settings was undertaken: (PS) (cmH_2_O)/(PEEP) (cmH_2_O): 0/10, 0/20, 10/4, 20/4, 10/10, 20/10. Ventilator settings were chosen to isolate the effects of mainly PEEP (two settings), mainly PS (two settings), and combinations of PEEP and PS (two settings). For technical reasons, isolated PS could not be administered without a PEEP of 4 cmH_2_O. Each change in ventilation pressures was followed by a 60-second pause to facilitate hemodynamic steady state before echocardiographic measurements were initiated.

Following the horizontal position, measurements at baseline and at all ventilator settings were repeated first in a 30-degree reverse-Trendelenburg position and, subsequently, in a 30-degree Trendelenburg position. Immediately prior to the Trendelenburg position, 1000 mL of pressurized isotonic saline was infused at a rate of approximately 200 mL/minute in a cubital vein in order to maximize preload. Fluid was not administered prior to the other positions.

### 2.3. Data Collection

All measurements were performed by the same experienced echocardiographer using a Vivid E9 echocardiography system fitted with an M5S phased array transducer (1.5–4.5 MHz) (GE Healthcare, Horten, Norway). Subjects were placed in the left lateral position for apical cardiac imaging. A second observer vouched for ventilator compliant respiration and ensured lack of air leakage from the mask before each measurement. 

Transmitral flow including early and atrial peak velocities, *E* and *A*, and *E*-deceleration time (*E*_dec_) was obtained in the 4-chamber view employing pulsed wave Doppler with the sample volume placed at the tips of the mitral valve leaflets. The early and atrial diastolic peak velocities, *e*′ and *a*′, were measured at the lateral mitral annulus using pulsed wave tissue Doppler. In the 5-chamber view, the velocity time integral (VTI) of the left ventricular outflow tract was quantified. All measurements were performed in sinus rhythm at end-expiration, and three consecutive cardiac cycles were stored for off-line analysis. The respiration tracing function was activated to ensure correct identification of the respiratory phase during off-line analysis.

### 2.4. Data Analysis

All echocardiographic data was analyzed by the same echocardiographer blinded from participant positioning and ventilator settings using dedicated software (Echopac, GE Healthcare, Horten, Norway). A second analysis on the whole dataset was performed by another person, blinded as well, for determination of interobserver agreement of image analyses. Measurements were averaged from triplicates. Cardiac output (CO) was calculated from the velocity time integral (VTI) of the left ventricular outflow tract (LVOT), the diameter of the LVOT and heart rate, as previously described [22]. 

### 2.5. Statistical Analyses

All repeated readings were analyzed using univariate ANOVAs for repeated measurements or Friedman's test where appropriate. This includes the overall influence of positioning at baseline and at individual ventilator settings (null hypothesis stating no effect of positioning), the effects of varying PPV (null hypothesis stating no effect of PPV), and the effects of positioning during PPV (null hypothesis stating equal positioning curves). At baseline, any two individual positions were compared with a paired Wilcoxon signed-rank test.

Interobserver bias was calculated as the mean difference in readings divided by the mean, expressed as a percentage and presented as mean bias with corresponding 95% limits of agreement (LoA) and 95% confidence interval (CI). A *P* value < 0.05 was considered statistically significant and *P* values stated are based on two-tailed analyses where applicable. STATA software (StataCorp LP, College Station, USA) was used in all analyses. Results are given as mean ± standard deviation.

## 3. Results

### 3.1. Effects of Changing Position

**Figure 2:**
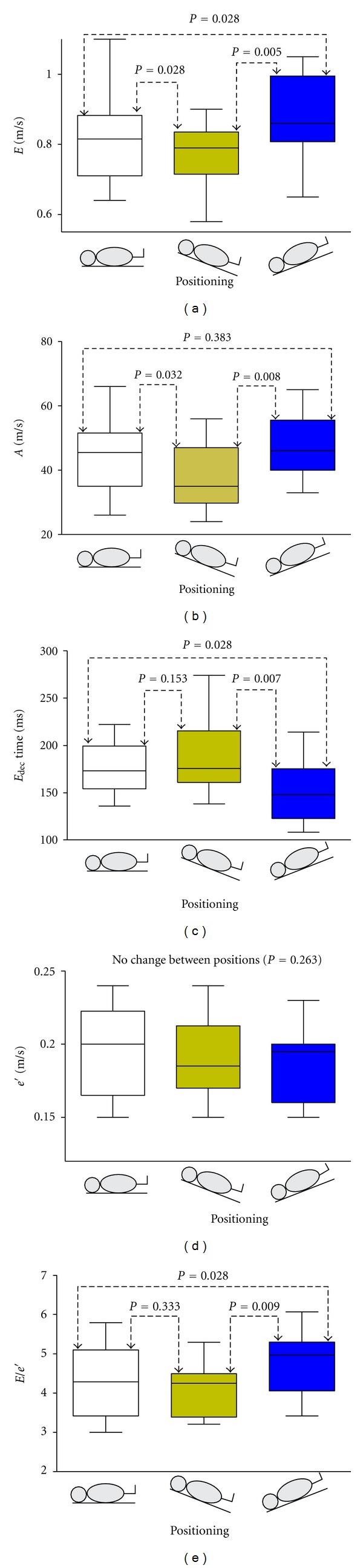
Effects of on- and off-loading during spontaneous respiration. Echocardiographic indices of diastolic function with subjects placed in varying positions during spontaneous respiration. *e*′: peak early diastolic velocity of the lateral mitral annulus. *E*: peak early transmitral flow. *A*: peak atrial transmitral flow. *E*_dec_: deceleration time of *E*. Data are presented as median, interquartile range (box) and range (whiskers). Graphs show independency of *e*′ to preload, whereas *E*, *E*_dec_, *A*, and *E*/*e*′ vary according to positioning.

**Figure 3:**
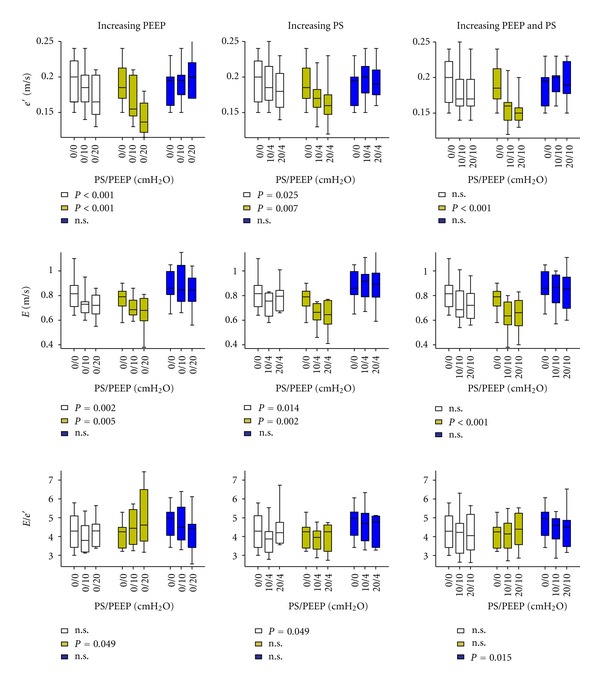
The influence of varying levels of positive pressure ventilation on diastolic echocardiographic indices. Effects of selective increases in either positive end-expiratory pressure (PEEP), pressure support (PS), or combinations of PEEP and PS on *e*′, *E* and the composite ratio, *E*/*e*′, in varying positions. *P* values reflect no influence of ventilator settings. n.s.: Not significant. Data are presented as median, interquartile range (box) and range (whiskers). Empty square: Horizontal position, yellow colored square: reverse-Trendelenburg position, blue colored square: Trendelenburg position.

During spontaneous ventilation, *e*′ was not dependent on positioning (*P* = 0.263), whereas *E*, *E*_dec_, *A*, and the composite value *E*/*e*′ proved dependent on positioning (All *P* < 0.020). *E* increased significantly in the Trendelenburg position and decreased in reverse-Trendelenburg in comparison with the horizontal position. *E*/*e*′ was significantly higher in the Trendelenburg position at baseline and insignificantly decreased in the reverse-Trendelenburg position (Figure 2). With application of PPV, *e*′, *E*, *E*/*e*′, *A*, and *E*_dec_ all proved overall dependent on positioning (all *P* values <0.008). For *e*′ and *E*, the effect was consistently significant at all individual ventilator settings (all *P* < 0.009) where the highest values were generated in the Trendelenburg position and the lowest values in the reverse-Trendelenburg position. This picture was not true for *E*/*e*′, where, despite overall significance, median values fluctuated within the range of 3.79 to 4.79 across positions and ventilator settings (Figure 3). *a*′ was independent of positioning (*P* = 0.109) throughout the study protocol.

### 3.2. Effects of Increasing PEEP

Both *e*′ and *E* decreased significantly with increasing PEEP in the horizontal- and reverse-Trendelenburg position, but no change was seen in the Trendelenburg position. The resulting effect on the composite ratio, *E*/*e*′, was diverging (Figure 3).

### 3.3. Effects of Increasing PS

Both *e*′ and *E* decreased significantly in the horizontal- and reverse-Trendelenburg positions with PS but remained unaffected in Trendelenburg position. *E*/*e*′ was not affected by increasing PS (Figure 3), except in the horizontal position.

### 3.4. Effects of Both PEEP and PS

A similar pattern was seen with application of both PEEP and PS. *e*′ was lowered in the horizontal- and reverse-Trendelenburg positions only. *E* decreased in the reverse-Trendelenburg position. Conversely, PEEP and PS affected *E*/*e*′ in the Trendelenburg position.

**Figure 4:**
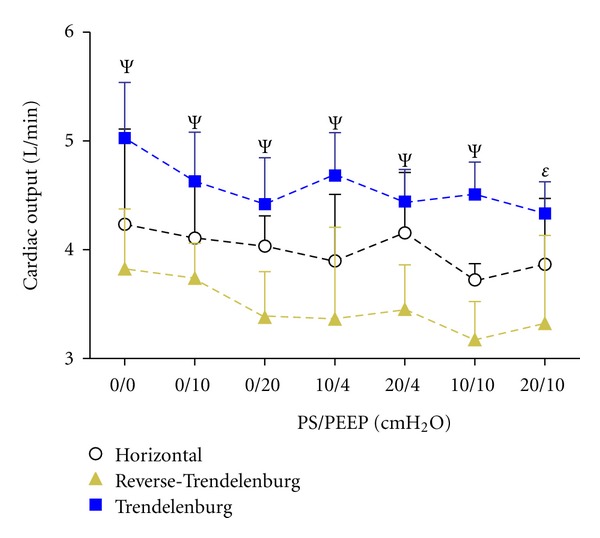
Effects of alternating positioning and varying positive pressure ventilation on cardiac output (CO). A clear association between positioning and CO was observed supporting the efficacy of our model of preload variation with positioning (*P* < 0.001). Ψ: *P* < 0.005 and *ε*: *P* < 0.05 for independence of positioning at individual ventilator settings. Data are presented as median and upper interquartile range.

Figure 4 depicts the significant influence of positioning on CO (*P* < 0.001). CO was consistently higher with increasing preload during spontaneous respiration and at all individual ventilator settings. Heart rates were as follows: horizontal position: 60.2  ±  7.9, reverse-Trendelenburg: 61.6  ±  7.2, and Trendelenburg: 60.3 ± 8.7. Mean arterial blood pressure (MAP) was affected by positioning (horizontal: 85.1 ± 5.6 mmHg, reverse-Trendelenburg: 84.5 ± 5.6 mmHg, Trendelenburg 86.8 ± 5.6 mmHg, *P* = 0.041), but was independent of ventilator settings (*P*-values > 0.105).

Interobserver bias was −1.8% (95% LoA −11.7%; 8.1%, 95% CI −2.3%; −1.3%) for tissue Doppler measurements (*e*′, *a*′), −0.9% (95% LoA −10.4%; 8.6%, 95% CI −1.3%; −0.5%) ± for transmitral flow values (*E*, *E*_dec_, *A*) and −1.9% (95% LoA −11.4%; 7.6%, 95% CI −2.6%; −1.2%) for VTI. Overall inter-observer bias was −1.4% (95% LoA −11.1%; 8.3%, 95% CI −1.7%; −1.1%). Mean time of obtaining all echocardiographic images was 161 s ±75 s from storage of the first image at each ventilator setting.

## 4. Discussion

This study shows that positioning changes affect echocardiographic indices of diastolic function. This was found for *E*, *A*, *E*_dec_, and *E*/*e*′, although the variation in median *E*/*e*′ was minimal and within two units. The Trendelenburg position elicited the clearest response which we attribute to the additional volume infusion administered prior to this position. The consistent influence of positioning and, hence, preload dependency of transmitral blood flow values is in accordance with previous studies [7, 8]. 

In regard to *e*′, an unambiguous dependency on positioning was found during PPV, whereas changes were insignificant during spontaneous respiration. Studies analyzing the influence of volume off-loading on *e*′ by means of hemodialysis or blood donation have yielded apparently conflicting results [9, 11, 13, 15, 23, 24]. However, a more detailed analysis reveals that studies removing less than 2 liters (or leading to a reduction in body weight of <2 kilograms) did not find *e*′ to be preload dependent and vice versa. Therefore, it seems *e*′ is fairly insensitive to smaller volume changes, but is reduced with substantial preload decrease. The results of our study are in line with this, as we believe the preload changes induced by shifting positions are reasonably small.

Early diastolic filling is determined by a complex interplay of multiple factors. These include preload, active relaxation of the cardiomyocytes and elastic recoil, which is the release of potential energy stored during the preceding contraction. Both *E* and *e*′ are reflections of early diastolic filling [25], and, among the mentioned factors, positioning mainly affects preload. Previous studies have shown that *E*/*e*′ correlates well with LV filling pressure [26]. As a consequence, *E* is more susceptible to change with altered filling pressure than *e*′. 

We found that increasing PEEP or PS influenced both *e*′ and *E* (Figure 3). The overall picture was a clear, negative effect in the reverse-Trendelenburg position and a modest effect when participants were placed horizontally. In the Trendelenburg position with a fluid bolus, neither *e*′ or *E* was affected by positioning.

The explanation for these findings may be found in the ways that PPV impacts hemodynamics. Firstly, PPV raises right atrial pressure with a consequent reduction in the pressure gradient for venous blood flow [27]. This attenuates right ventricular (RV), preload and the effect is transmitted to the LV a few heart beats later. In addition, PPV-induced pulmonary hyperinflation increases pulmonary vascular resistance [28] further reducing LV preload. A substantial increase in pulmonary vascular resistance will, with adequate preload, cause RV dilatation, a septal shift and reduction in LV contraction due to interventricular dependence [29].

Secondly, PPV reduces LV transmural pressure resulting in a reduction in afterload. This is contingent on adequate baroreceptor feedback which was confirmed in the present study, as MAP was independent of ventilator settings. The effect of transmural pressure reduction is a more powerful contraction with storage of more potential energy to be released in the following diastole as elastic recoil.

Hence, PPV affects both LV preload and contractility. These factors influence *E* and *e*′ in opposite directions. We have shown that the net result depends on the participants positioning. A previous animal study has shown that a PEEP = induced lowering of CO may be restored to normal values with volume overload [30]. A different study on humans with chronic heart failure showed that the application of PEEP (5 cmH_2_O) resulted in lowering of CO if pulmonary wedge pressure was below 11 mmHg, whereas CO was increased with PEEP if pulmonary wedge pressure was higher [31]. Our findings parallel these studies, as the Trendelenburg position in combination with a fluid bolus cancelled out the effect of PPV. We therefore speculate that, in regard to the determining factors of *E* and *e*′, PPV predominantly alters preload, but this may be nullified if RV filling is sufficient. 

A few studies have addressed the influence of PEEP on *e*′ and found no significance difference as PEEP was applied. However, PEEP levels at 10-11 cmH_2_O were lower than the pressure levels of our study, and the study populations were children aged 3 months to 12 years and patients with ejection fraction <40%, respectively [20, 21]. These factors may explain the differing findings.

The physiologic effects of PPV may also account for the shift towards positioning dependence of *e*′ witnessed as PPV was applied. PPV reduces end-diastolic volume as the transmural pressure gradient drops [29]. Consequently, end-diastolic myocardial fiber length is shortened. At short myocardial fiber lengths, the LV may be more sensitive to volume changes. A given change in load may cause an increased myocardial lengthening rate during diastolic passive filling at short myocardial fiber lengths, but not at long fiber lengths in analogy to the Frank-Starling relationship.

To our knowledge, this study is the first to address PPV in both respiratory phases and to report its influence on tissue Doppler echocardiographic indices related to diastolic function. We did not incorporate other indices of diastolic function such as pulmonary venous flow or flow propagation velocity, as these measures have demonstrated poor feasibility and reproducibility [26, 32]. These impediments are likely augmented in clinical settings that include PPV. 

### 4.1. Study Limitations

The participants in our study were all healthy with normal cardiac function and are not representative of hospital patients. All participants had normal diastolic function with *E*/*e*′ in the normal range. Current guidelines propose a cut-off value of >8 before considering impaired relaxation properties [5]. All participants stayed below this limit throughout the protocol and, although variation in *E*/*e*′ was statistically significant, the physiological and clinical implications of changes under the cut-off value remain poorly understood and have little clinical impact. *E*/*e*′ is regarded as a measure of LV filling pressure but cannot stand alone and should be part of an integrated approach [33].

A large proportion of the elderly population or patients in intensive care setting have diastolic compromise from arterial hypertension, sepsis, or other causes. Our findings of echocardiographic preload and PPV sensitivity do not necessarily apply to patients with diastolic dysfunction, and one can speculate that the presence of impaired relaxation reduces the alterations seen in *E* or *e*′ with preload shifts or PPV.

We measured *e*′ at the lateral mitral annulus, but not at the medial mitral annulus. Current guidelines recommend that *e*′ is averaged from these two sites [5], at least in patients with regional dyskinesia. Studies have shown that the medial mitral annulus is more sensitive to preload change [15, 34]. Inclusion of the medial *e*′ may have shifted our results towards a more pronounced effect of positioning and varying PPV. 

In addition, we chose a fixed sequence of ventilator settings in order to optimize participant compliance with PPV, although this leaves the potential for a carry-over effect. 

## 5. Conclusion

*E* was dependent on positioning during spontaneous respiration and PPV. Conversely, *e*′ proved independent of positioning changes during spontaneous respiration, but elicited a systematic sensitivity to positioning with application of PPV. 

Increasing PEEP, PS, or both PEEP and PS decreased *E* and *e*′ in the reverse-Trendelenburg position. The effect was less pronounced, although significant, in the horizontal position and absent in the Trendelenburg position supplemented with a fluid bolus. 

This study questions the feasibility of echocardiographic evaluation of diastolic function in otherwise healthy subjects exposed to acute preload changes or PPV.

## 6. Key Messages

- *E*  and *e*′ proved unequivocally dependent on positioning during positive pressure ventilation.
- The effects of positive pressure ventilation on tissue Doppler measures of diastolic function are pronounced in the reverse-Trendelenburg- and horizontal positions, but disappear at high preload in the Trendelenburg position with a fluid bolus.

## List of Abbreviations

*A*: Diastolic transmitral flow generated by atrial contraction
*a*′: Diastolic peak velocity of the lateral mitral annulus generated by atrial contraction
CI: Confidence interval
CO: Cardiac output
*E*: Early diastolic transmitral flow
*E*_dec_: Deceleration time of the E-wave
*e*′: Early diastolic peak velocity of the lateral mitral annulus
LoA: Limits of agreement
LV: Left ventricle
LVOT: Left ventricular outflow tract
MAP: Mean arterial pressure
PEEP: Positive end-expiratory pressure
PPV: Positive pressure ventilation
PS: Pressure support
RV: Right ventricle
VTI: Velocity time integral.
